# A Comprehensive Network Pharmacology-Based Strategy to Investigate Multiple Mechanisms of HeChan Tablet on Lung Cancer

**DOI:** 10.1155/2020/7658342

**Published:** 2020-05-30

**Authors:** Zhenjie Zhuang, Qianying Chen, Cihui Huang, Junmao Wen, Haifu Huang, Zhanhua Liu

**Affiliations:** ^1^Guangzhou University of Chinese Medicine, Guangzhou, China; ^2^Shenzhen Hospital of Guangzhou University of Chinese Medicine, Shenzhen, China; ^3^Department of Oncology, The First Affiliated Hospital of Guangzhou University of Chinese Medicine, Guangzhou, China

## Abstract

**Background:**

HeChan tablet (HCT) is a traditional Chinese medicine preparation extensively prescribed to treat lung cancer in China. However, the pharmacological mechanisms of HCT on lung cancer remain to be elucidated.

**Methods:**

A comprehensive network pharmacology-based strategy was conducted to explore underlying mechanisms of HCT on lung cancer. Putative targets and compounds of HCT were retrieved from TCMSP and BATMAN-TCM databases; related genes of lung cancer were retrieved from OMIM and DisGeNET databases; known therapeutic target genes of lung cancer were retrieved from TTD and DrugBank databases; PPI networks among target genes were constructed to filter hub genes by STRING. Furthermore, the pathway and GO enrichment analysis of hub genes was performed by clusterProfiler, and the clinical significance of hub genes was identified by The Cancer Genome Atlas.

**Result:**

A total of 206 compounds and 2,433 target genes of HCT were obtained. 5,317 related genes of lung cancer and 77 known therapeutic target genes of lung cancer were identified. 507 unique target genes were identified among HCT-related genes of lung cancer and 34 unique target genes were identified among HCT-known therapeutic target genes of lung cancer. By PPI networks, 11 target genes AKT1, TP53, MAPK8, JUN, EGFR, TNF, INS, IL-6, MYC, VEGFA, and MAPK1 were identified as major hub genes. IL-6, JUN, EGFR, and MYC were shown to associate with the survival of lung cancer patients. Five compounds of HCT， quercetin, luteolin, kaempferol, beta-sitosterol, and baicalein were recognized as key compounds of HCT on lung cancer. The gene enrichment analysis implied that HCT probably benefitted patients with lung cancer by modulating the MAPK and PI3K-Akt pathways.

**Conclusion:**

This study predicted pharmacological and molecular mechanisms of HCT against lung cancer and could pave the way for further experimental research and clinical application of HCT.

## 1. Introduction

Lung cancer is the leading cause of cancer-related death worldwide with approximately two million diagnosed cases every year [1]. Lung cancer is divided mainly into small-cell lung cancer (SCLC) and non-small-cell lung cancer (NSCLC), and NSCLC mainly includes lung squamous cell carcinoma (LUSC), lung adenocarcinoma (LUAD), and large cell carcinoma (LCC). The prevalence of NSCLC occupies 85% of lung cancer cases [2]. Although significant development has been made in research and precision medicine in the past few decades, yet 5-year survival in lung cancer patients ranges from 4 to 17% [3]. Traditional Chinese medicine is an important part of complementary and alternative medicine which has built up valuable experience in treating cancer [4]. Approved by the China National Medical Products Administration (approval number Z44022453), HeChan tablet (HCT) is a traditional Chinese medicine preparation widely used for treating lung cancer in China. HCT is composed of nine herbs including *Pinellia ternate* (Sheng Ban Xia (SBX)), *Bufo bufo gargarizans Cantor* (Gan Chan Pi (GCP)), *Ranunculus ternatus Thunb* (Mao Zhao Cao (MZC)), *Panax ginseng C. A. Mey* (Ren Shen (RS)), *Asparagus cochinchinensis* (Tian Dong (TD)), *Lepidium apetalum Willd* (Ting Li Zi (TLZ)), *Agrimonia pilosa Ledeb* (Xian He Cao (XHC)), *Houttuynia cordata Thunb* (Yu Xing Cao (YXC)), and *Fritillaria thunbergii Miq* (Zhe Bei Mu (ZBM)).

It is found that HCT could induce apoptosis of human lung adenocarcinoma A549 cells by the inhibition of EGFR gene transcription in vivo study [5]. Moreover, clinical studies indicated that HCT combined chemotherapy could prolong progression-free survival, improve disease symptoms, and reduce adverse events of anticancer treatment [6–8]. As HCT consists of chemical compounds which may regulate a considerable number of target genes potentially, it is necessary to carry out further research on HCT to reveal pharmacological mechanisms.

**Figure 1:**
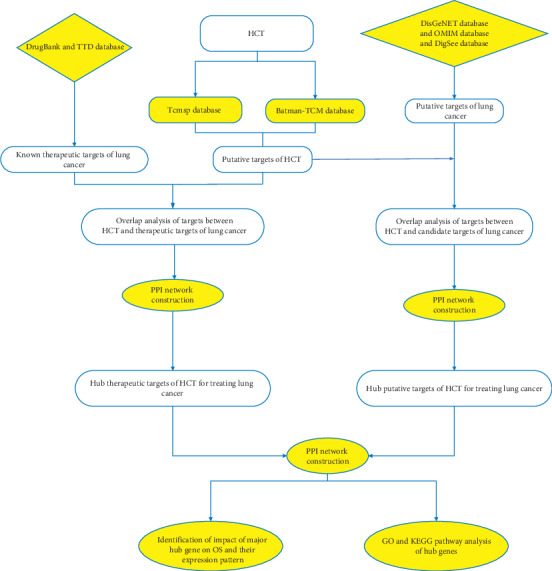
The framework of this study based on network pharmacology strategies for deciphering pharmacological mechanisms of HCT acting on lung cancer. HCT: HeChan tablets; TCMSP: Traditional Chinese Medicine Systems Pharmacology database; BATMAN-TCM: Bioinformatics Analysis Tool for Molecular mechANism of Traditional Chinese Medicine; OMIM: Online Mendelian Inheritance in Man; KEGG: Kyoto Encyclopedia of Genes and Genomes; GO: Gene Ontology; PPI: protein-protein interaction; OS: overall survival; DFS: disease-free survival.

Network pharmacology is a comprehensive and systematic approach to uncover interactions of multi-ingredient medicines and their targets [9]. It is broadly applied to the molecular design of multitarget drug based on analysis of biological systems and selected specific signal nodes in networks [10]. With the practice of network pharmacology, the study pattern of traditional Chinese medicine has been transformed into the mode of “multicomponent, multigene and multitarget” [11]. This study aims at investigating potential active ingredients, target genes, and underlying pharmacological mechanisms of HCT in the treatment of lung cancer based on a comprehensive network pharmacology-based strategy (Figure 1).

## 2. Method and Materials

### 2.1. Chemical Ingredients and Targets Database Construction

The chemical ingredients and target genes of 9 herbs in HCT were screened on Traditional Chinese Medicine Systems Pharmacology database (TCMSP) and Bioinformatics Analysis Tool for Molecular mechANism of Traditional Chinese Medicine (BATMAN-TCM). TCMSP contains 499 herbs registered in the Chinese Pharmacopoeia with 29,384 ingredients, 3,311 targets, and 837 associated diseases and it exhibits the drug-gene interaction based on both experiment and prediction [12]. Oral bioavailability ≥30% and drug-likeness ≥0.18 were set as thresholds according to the guidance of TCMSP. Additionally, gene symbols of the screened genes on TCMSP were verified by UniProt [13].

BATMAN-TCM is an online platform integrating database resources including Kyoto Encyclopedia of Genes and Genomes (KEGG), DrugBank, and Therapeutic Target Database (TTD). It provides the drug-gene interaction mainly based on a similarity-based target prediction method and is practical to systematically reveal mechanisms of traditional Chinese medicine [14]. The data were collected from BATMAN-TCM and loaded into R software (version 3.6.1), and then targets which had higher scores than the mean score were obtained. Thereafter, the data extracted from TCMSP and BATMAN-TCM were combined to construct the chemical ingredients and targets database.

### 2.2. Collection of Related Genes of Lung Cancer

Lung cancer-related genes were collected from DisGeNET (version 6.0 updated on July 2019), Online Mendelian Inheritance in Man (OMIM updated on September 6, 2019), and DigSee (updated on June 2016).

Based on integrative data from expert curated repositories, GWAS catalogues, animal experiments, and the scientific literature [15], DisGeNET is a discovery platform containing one of the largest public available collections of genes and variants associated with human diseases. Gene-disease association files including “curated gene-disease associations,” “be free gene-disease associations,” “all gene-disease associations,” and “all gene-disease-pmid associations” were downloaded and loaded into R software (version 3.6.1) to obtain lung cancer-related genes. DigSee is a search engine developed to find association between gene and cancer through biological events [16]. The keyword, “lung neoplasm,” was searched in DigSee for related genes of lung cancer. OMIM is a curated database which provides over 15,000 genes information derived from the published peer-reviewed biomedical literature [17]. After registered on OMIM, the file named “morbidmap” which included information of genes and their relative diseases were downloaded and loaded into R software (version 3.6.1) to obtain lung cancer-related genes. Target genes shared by HCT and lung cancer were collected as putative targets of HCT on lung cancer.

### 2.3. Collection of Known Therapeutic Target Genes Acting on Lung Cancer

The collection of known therapeutic target genes acting on lung cancer was completed by DrugBank (version 5.1.4, released on 2019-07-02) and Therapeutic Target Database (TTD, updated on September 15, 2017). DrugBank contains 13,438 drug entries including 2,617 approved small molecule drugs, 1,345 approved biotech (protein/peptide) drugs, 130 nutraceuticals, and over 6,335 experimental drugs and their targets [18]. Data from DrugBank were downloaded and then analyzed by package *DrugBankR* in R software (version 3.6.1) [19]. The selection of target genes was conducted among drugs approved by the Food and Drug Administration for the treatment of lung cancer. TTD provides information about 34,019 drugs, 3,101 targets, related diseases, and pathways [20]. The TTD target genes information was downloaded, and therapeutic targets of lung cancer were identified by R software (version 3.6.1).

### 2.4. Data Cleaning

The data cleaning was performed by package *dplyr* [21] and package *tidyr* [22] in R software (version 3.6.1) after preparing the data of target genes of HCT and lung cancer. The compounds and target genes of HCT from TCMSP and BATMAN-TCM were combined, and duplicate items were removed. Afterwards, the number of common target genes of herbs in HCT, related target genes of lung cancer and HCT, and the known therapeutic target genes of lung cancer and HCT was calculated. The shared genes between HCT-related genes of lung cancer and HCT-known therapeutic target genes of lung cancer were identified to construct protein-protein interaction (PPI) network.

### 2.5. Protein-Protein Interaction (PPI) Network Construction and Identification of Hub Targets

The interaction between HCT and putative target genes of lung cancer was generated by STRING 11.0 (https://string-db.org/, updated on January 19, 2019) [23]. To assure the robustness of the result, the high confidence of interaction was applied when the interaction score was higher than or equal to 0.7. Then protein networks were constructed and analyzed by Cytoscape 3.72 [24]. A node would be identified as a hub in the network if its degree was over two times that of the median [25–28]. Subsequently, hub genes were filtered from two networks and input into STRING again to explore the protein interaction between putative and the known therapeutic target genes of lung cancer.

### 2.6. Gene Ontology (GO) and KEGG Pathway Enrichment Analysis

The *clusterProfiler* [29] is a functional package in R software used for calculating the KEGG pathway and analysing GO ontology including biological processes (BP), molecular function (MF), and cellular component (CC). Based on multiple bioconductor annotation resources and R packages, the package *clusterProfiler* was dynamically updated and widely used for bioinformatics analysis. The cutoff of *P* value was set to 0.01, and terms with *P* value less than or equal to 0.01 were included. The false discovery rate was applied for *P* value adjustment.

### 2.7. Identification of Clinical Significance of Hub Genes

The transcriptome profiling data of lung cancer from The Cancer Genome Atlas (TCGA) (https://www.cancer.gov/tcga.) were applied to identify the clinical significance of the hub genes filtered from the putative target genes-known therapeutic target genes protein network. As a milestone of cancer genomics program, TCGA matches normal samples covering 33 cancer types and provides molecular characterizations of more than 20,000 primary cancers. As TCGA only offers data of LUSC and LUAD, the analysis was conducted based on these pathological patterns of lung cancer. Transcriptome profiling data of 1,037 NSCLC tissues (LUAD: 535; LUSC: 502) and 108 normal paracarcinoma tissues (LUAD: 59; LUSC: 49) from 1,014 NSCLC patients (LUAD: 513; LUSC: 501) who have complete clinical data were downloaded by an R package named *TCGAbiolinks* [30]. The gene expression data were standardized by an R package named *DESeq2* [31] before the analysis. Then, the univariate and multivariate survival analyses were performed to identify the survival impact of hub genes on patients with NSCLC by the log-rank test and Cox's proportional hazards regression, respectively. In addition, we applied an R package named *survminer* [32] to draw the survival curve and to calculate the cutoff expression value of the hub genes. Moreover, the expression pattern of hub genes that had an impact on the survival of NSCLC patients would be further analyzed by R software (version 3.6.1). The normality test on hub gene expression data was performed by the Shapiro–Wilk test. Then, the statistical significance between the cancer group and the normal group would be checked by a *t*-test, if the expression data reached the normality. Otherwise, the statistical significance between the two groups would be checked by Wilcoxon test instead. R package named *ggplot2* [33] was used for drawing the boxplot of the expression pattern of hub genes.

## 3. Result

### 3.1. Target Genes Analysis between HCT and Lung Cancer

A total of 206 compounds of 9 herbs contained in HCT were collected. In total, 2,433 genes were predicted as the putative targets of HCT (BX: 415; GCP: 203; MZC: 53; RS: 516; TD: 330; TLZ: 199; XHC: 205; YXC: 371; ZBM: 141). Detailed information about the putative targets of HCT is provided in Supplementary file 1. The quantity of common putative target genes among herbs is shown in Table 1, indicating that these herbs may have interactions in the treatment of lung cancer.

The number of genes obtained from DisGeNET, DigSee, and OMIM was 4,251, 2,798, and 67, respectively. With the removal of duplicated genes, 5,317 genes were identified as related genes of lung cancer. Besides that, 30 known therapeutic target genes of lung cancer were obtained from DrugBank and 54 were obtained from TTD. 54 genes were identified after removing the duplicated genes.

Common target genes were identified among target genes of HCT, related genes of lung cancer, and known therapeutic target genes of lung cancer. The quantity of shared targets between herbs and HCT-related genes of lung cancer and the number of shared targets between herbs and HCT-known therapeutic targets of lung cancer are shown in Table 2. 507 unique target genes were identified among HCT-related genes of lung cancer and 34 unique target genes were identified among HCT-known therapeutic target genes of lung cancer. Detailed information is provided in Supplementary file 2 and Supplementary file 3. These target genes were collected for the subsequent analysis.

### 3.2. PPI Network Analysis

**Figure 2:**
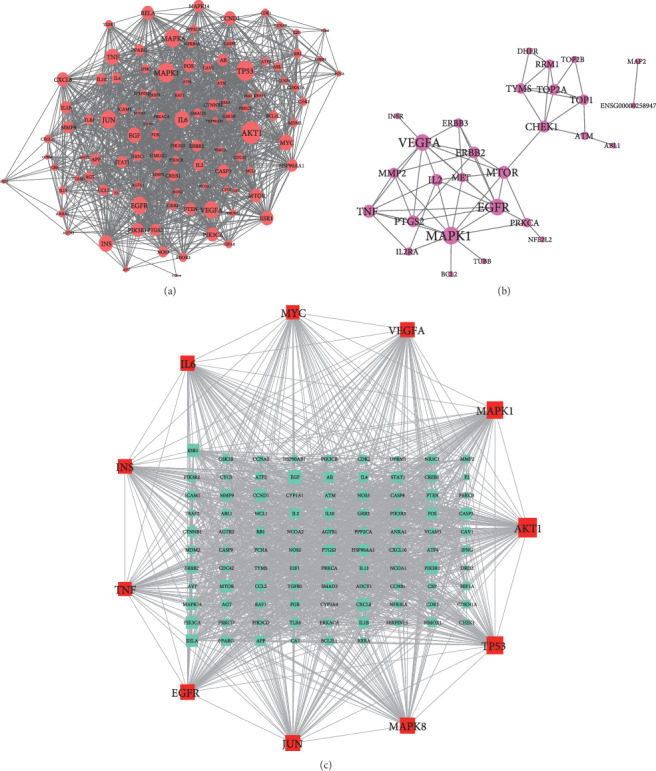
PPI network analysis. (a) Protein-protein interaction network of common genes of HCT-related genes of lung cancer. Note: round red nodes stand for hub targets of PPI network of common gene targets between ingredients contained in HCT and related genes of lung cancer. The greater the degree of the node was, the greater the size of the node was. (b) Protein-protein interaction network of common genes of HCT-known therapeutic gene targets of lung cancer. Note: round purple nodes stand for hub targets of PPI network of common gene targets between ingredients contained in HCT and known therapeutic gene targets of lung cancer. The greater the degree of the node was, the greater the size of the node was. (c) PPI interaction network of hub genes between HCT-related genes of lung cancer PPI network and HCT-known therapeutic gene targets and of lung cancer PPI network. Note: square red nodes stand for major hub targets of PPI network. Square cyan nodes stand for the other hub genes. The greater the degree of the node was, the greater the size of the node was.

The PPI network of common target genes between HCT and related genes of lung cancer consisted of 497 nodes and 4,231 edges. Ten genes were removed from this network for the failure of reaching the filter threshold value of STRING. The twofold median value of node degree in this network was 24. After setting the cutoff point to 24, 107 nodes were identified as hub genes. Detailed information is provided in Supplementary file 4. Due to the bulkiness of this network, the network showing interactions among 107 hub genes is presented instead (Figure 2(a)).

In addition, the PPI network of common target genes between HCT-known therapeutic targets of lung cancer contained 28 nodes and 61 edges (Figure 2(b)). The cutoff value of the hub gene in this network was 8, and three genes were identified as hubs. Detailed information is provided in Supplementary file 5. These genes were epidermal growth factor receptor (EGFR), vascular endothelial growth factor (VEGFA), and mitogen-activated protein kinase-1(MAPK1). The overlap of these three genes in hub genes of HCT-related genes of lung cancer suggested that these hub genes might be more important in the treatment of lung cancer. In order to explore the interaction between HCT-related hub genes of lung cancer and HCT-known therapeutic hub genes of lung cancer, another PPI network was constructed. The condition of network construction was also set to high confidence (interaction score ≥ 0.7) and the disconnected nodes were excluded in the network. This PPI network consisted of 107 nodes and 1,479 edges, and the twofold median value of node degree was 48 (Figure 2(c)). Filtered by 48, a total of 11 genes were identified as major hub genes of HCT on lung cancer (detailed information of these 11 hub genes is shown in Table 3). Thus, these hub genes were used for revealing the potential mechanisms of HCT on lung cancer. Detailed information is provided in Supplementary file 6.

### 3.3. Analysis of Potential Mechanisms of HCT in the Treatment of Lung Cancer

**Figure 3:**
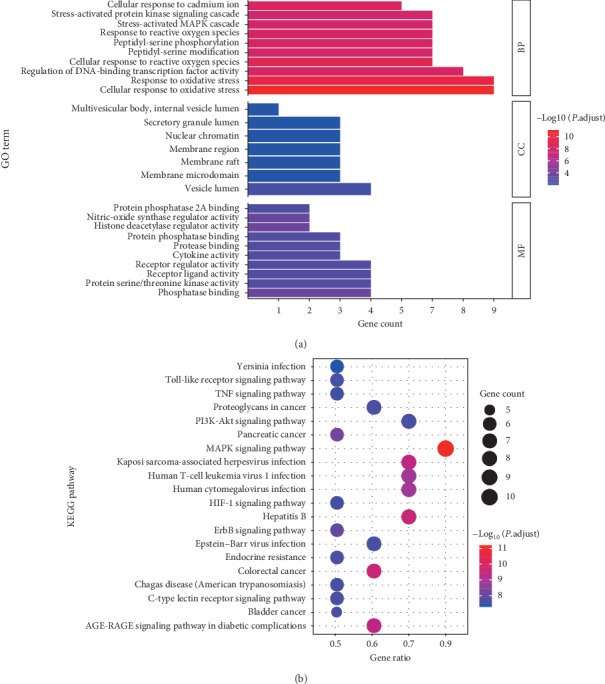
Gene Ontology (GO) and KEGG pathway enrichment analysis. (a) Main Gene Ontology terms enriched by major hubs from clusterProfiler. The top 10 terms of BP, MM, and CC measured by adjusted *P* value were selected to demonstrate. Note: the color of terms turned from blue to red. The redder the bar was, the smaller the adjusted *P* value was. (b) KEGG pathway enriched by major hubs from clusterProfiler. The top 20 terms of pathways measured by adjusted *P* value were selected to demonstrate. Note: the color of terms turned from blue to red. The redder the bubble was, the smaller the adjusted *P* value was. BP: biological processes; MF, molecular function; CC: cellular component.

**Figure 4:**
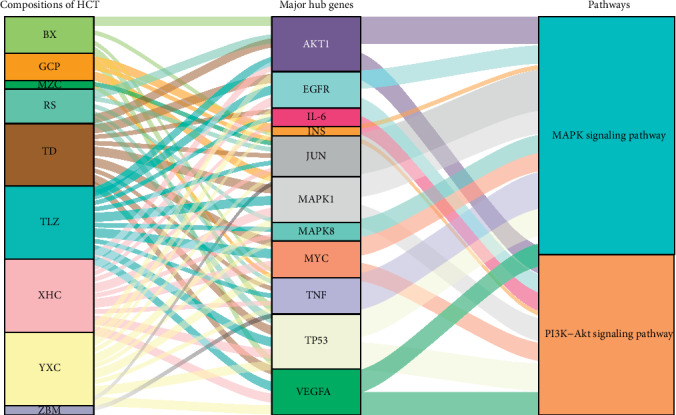
Sankey diagram of interaction between herbs of HCT, major hub genes, and two main pathways. Note: the herbs, major hub genes, and main pathways were displayed in different colors. The height of the rectangle and the width of the connecting line were positively correlated with the number of other rectangles they connected.

**Figure 5:**
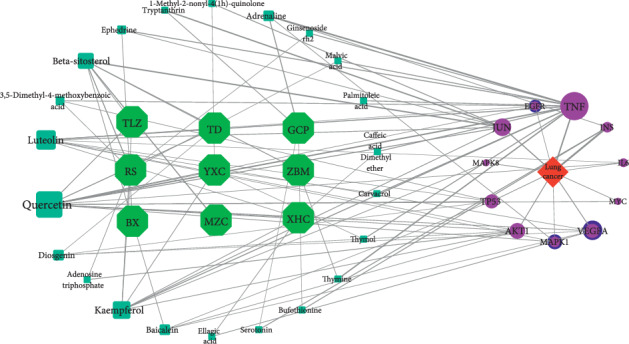
The interaction network of herbs of HCT-compounds-lung cancer-major hubs. Note: the green hexagon nodes stand for herbs of HCT, and the cyan square nodes stand for compounds of herbs. The red square node stands for the disease, and the circular nodes stand for the major hub genes. The greater the degree of the hub genes and compound was, the greater the size of the nodes was.

(1) Result of bioinformatics annotation: the major hub genes in PPI network were input into *clusterProfiler* for GO and KEGG analysis. 912 GO terms were ascertained, including 874 BP terms, 31 MF terms, and 7 CC terms. The major BP included stress-activated protein kinase signaling cascade, stress-activated MAPK cascade, and response to reactive oxygen species. The major CC included secretory granule lumen, nuclear chromatin, and membrane region. The major MF included protein phosphatase 2A binding, nitric-oxide synthase regulator activity, and histone deacetylase regulator activity. The top ten terms of each category are shown by bar chart (Figure 3(a)). In total, 172 KEGG pathways were identified. The MAPK signaling pathway and PI3K-Akt signaling pathway were two major pathways with the highest gene ratios and the lowest*P*values; therefore, they might play important roles in HCT on lung cancer. The top twenty KEGG pathway terms are shown by advanced bubble chart (Figure 3(b)). In addition, the relationship between herbs of HCT, hub genes, and major pathways was visualized by the Sankey diagram (Figure 4). These results indicated that HCT might exert its therapeutic effect on lung cancer through multiple biological processes and pathways. (2) The relationship between HCT and hub genes: the HCT-major hub genes-disease network was constructed to explore and analyse potential mechanisms of HCT on lung cancer (Figure 5). This network contained 43 nodes and 92 edges. The degree order of major hub targets from the greatest to the smallest was as follows: TNF (degree: 14) > JUN (degree: 8) > AKT1 (degree: 7) > VEGFA (degree: 6) > TP53 (degree: 4) = INS (degree: 4) = MAPK1 (degree: 4) = IL-6 (degree: 4) = MAPK8 (degree: 4) = EFGR (degree: 4) = MYC (degree: 4). Moreover, the top five compounds with the greatest degrees in the network were quercetin (degree: 13), luteolin (degree: 9), kaempferol (degree: 8), beta-sitosterol (degree: 7), and baicalein (degree: 4), suggesting that these compounds might play important roles in lung cancer treatment.

### 3.4. Clinical Significance of the Major Hub Targets

To clarify the correlation between major hub genes and the prognosis of lung cancer, a search of lung cancer data from TCGA was performed to determine their impacts on the prognosis of lung cancer. The expression pattern of the major hub genes and their impacts on the survival of 1,014 NSCLC patients (LUAD: 513; LUSC: 501) were identified one by one and significant outcomes were presented.

**Figure 6:**
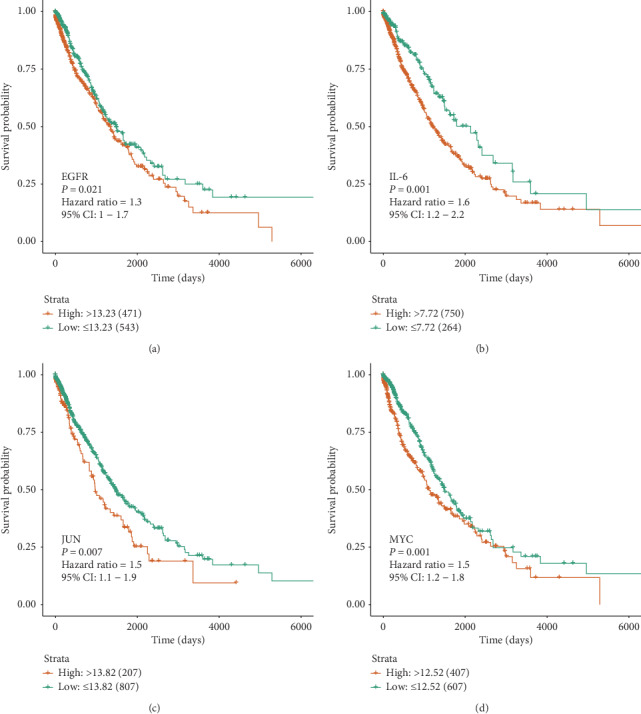
Univariate survival analysis of the major hub genes. (a, b) Kaplan–Meier curves of overall survival of non-small-cell lung cancer (NSCLC) patient with EGFR/IL-6. (c, d) Kaplan–Meier curves of overall survival of non-small-cell lung cancer (NSCLC) patient with JUN/MYC.

**Figure 7:**
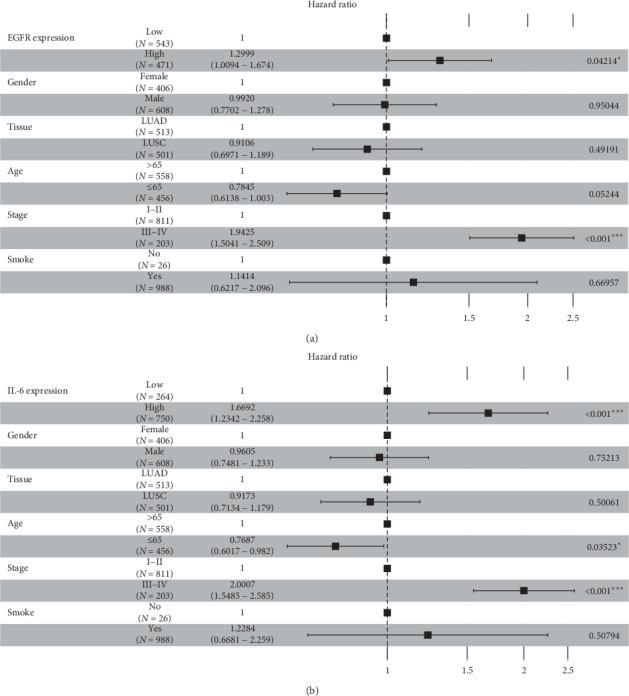
Forest plot of multivariate survival analysis of (a) EGFR and (b) IL-6.

**Figure 8:**
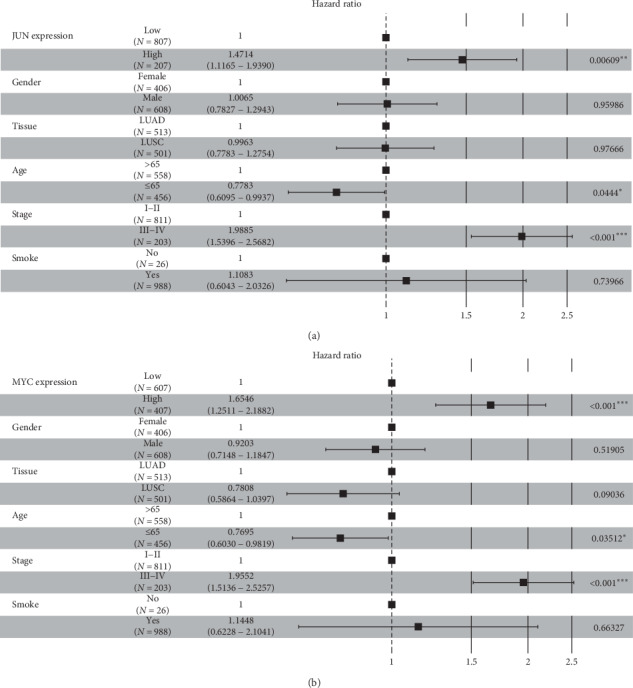
Forest plot of multivariate survival analysis of (a) JUN and (b) MYC.

As a result, among 11 major hub genes, totally 4 genes, EGFR, IL-6, JUN, and MYC were found to have an impact on the overall survival (OS) of NSCLC patients. In the univariate survival analysis, NSCLC patients with the high expression level of EGFR had a poorer OS than the low expression group (cutoff expression value: 13.23; *P* value*:* 0.021; HazardHR: 1.3; 95% CI: 1–1.7), and this result was quite similar to the expression level of IL-6 (cutoff expression value: 7.72; *P* value*:* 0.001; HR: 1.6; 95% CI: 1.2–1.7) (Figures 6(a) and 6(b)). In addition, the high expression level of JUN was associated with a poor OS of NSCLC patients (cutoff expression value: 13.82; *P* value: 0.007; HR: 1.5; 95% CI: 1.1–1.9), and the high expression level of MYC was associated with a poor OS (cutoff expression value: 12.52; *P* value: 0.001; HR: 1.5; 95% CI: 1.1–1.9) (Figures 6(c) and 6(d)). To further identify the impacts of these four major hub genes on OS of NSCLC patients, the multivariate survival analysis was performed to adjust factors including gender, tissue types, age, cancer stage, and smoke status. Among 1,014 NSCLC patients, 406 patients were female and 608 patients were male; 558 patients were over 65 years of age and 456 patients were 65 years old or below; 811 patients had a disease at stage I-II, while 203 patients were at stage III-IV; 988 patients had a history of smoking, only 26 patients were never smokers. Sample sizes and the cutoff expression value of every gene were consistent with the univariate survival analysis. Interestingly, we also found that high expression levels of these four major genes were correlated with the poor OS of NSCLC patients: EGFR (HR: 1.2999; 95% CI: 1.0094–1.674; *P* value: 0.4214); IL-6 (HR: 1.6692; 95% CI: 1.23432–2.258; *P* value <0.001); JUN (HR: 1.4714; 95% CI: 1.1165–1.9390; *P* value: 0.00609); MYC (HR: 1.6546; 95% CI: 1.2511–2.1882; *P* value <0.001) (Figures 7(a), 7(b), 8(a), and 8(b)). The high expression levels of these four genes might be independent risk factors for the survival of NSCLC patients.

**Figure 9:**
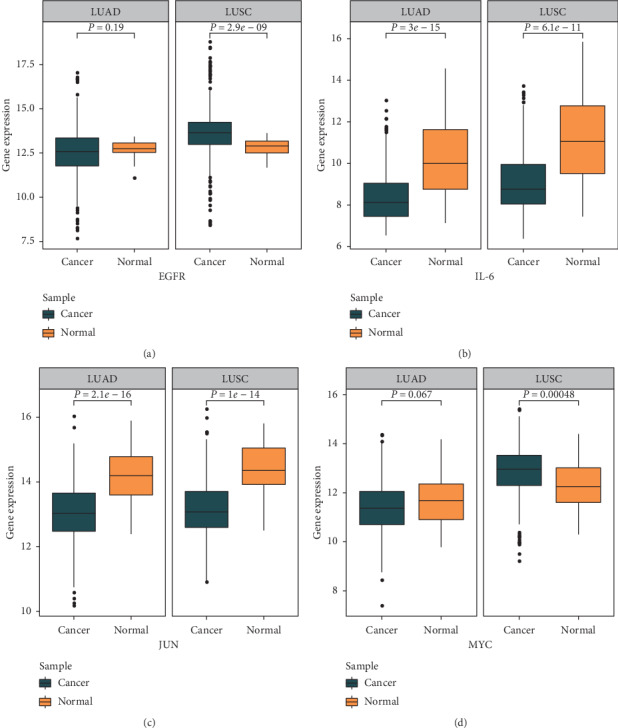
Expression pattern of the major hub genes in non-small-cell lung cancer. (a, b) Expression patterns of EGFR/IL-6 in LUAD and LUSC. (b, d) Expression patterns of JUN/MYC in LUAD and LUSC.

Furthermore, expression patterns of these four major genes were identified among 1,037 NSCLC tissues (LUAD: 535; LUSC: 502) and 108 normal adjacent tissues (LUAD: 59; LUSC: 49). Due to the nonnormality of the expression data, the Wilcoxon test was used to check their expression patterns between cancer and normal groups. The expression of EGFR in LUAD-tissue group was lower than the normal with no statistical significance (*P* value: 0.19), while the expression of EGFR in LUSC-tissue group was higher than the normal with statistical significance (*P* value <0.001) (Figure 9(a)); IL-6 had lower expression levels in both LUAD and LUSC groups than in the normal (*P* value <0.001; *P* value <0.001), and this expression pattern was similar to JUN (*P* value <0.001; *P* value <0.001) (Figures 9(b) and 9(c)); MYC also had a lower expression level in LUAD-tissue group than the normal group with no statistical significance (*P* value: 0.067), but it had higher expression level in LUSC-tissue group than the normal (*P* value <0.001) (Figure 9(d)). These findings may provide background knowledge about the expression pattern of these major hub genes for further studies.

To sum up, this study showed that the high expression level of EGFR, IL-6, JUN, and MYC was associated with worse OS of NSCLC patients according to results of univariate survival analysis and multivariate survival analysis. EGFR and MYC had significantly higher expression levels in LUSC tissues, while IL-6 and JUN had significantly lower expression levels in both LUSC and LUAD tissues. In conclusion, HCT may benefit lung cancer patients and exert its therapeutic effect on lung cancer by regulating the expression of major hub genes.

## 4. Discussion

Despite great strides in chemotherapy, radiotherapy, targeted therapy, and immunotherapy, lung cancer remains to be one of the leading causes of death from cancer worldwide and a major public health problem with an unfavorable prognosis and a poor 5-year overall survival [3]. To find new agents on lung cancer is of importance for clinical treatment. HCT has been prescribed to patients with lung cancer for decades [34]. Although published studies have shown that HCT induced the apoptosis of cancer cells in vitro and improved the condition of lung cancer patients [6,7], yet no available literature has elucidated underlying mechanisms of HCT on lung cancer adequately; therefore, further research is required to reveal unexplored mechanisms. To the best of our knowledge, this is the first comprehensive and cross-database network pharmacology analysis on mechanisms of HCT in the treatment of lung cancer.

By network pharmacology, the identification of key compounds of traditional Chinese medicineformulations will facilitate further research on pharmacological mechanisms offormulations. In HCT-major hub genes-disease network, quercetin, luteolin, kaempferol, beta-sitosterol, and baicalein were compounds with top five degrees among major hub disease genes. Quercetin could exert anticancer effects in lung cancer cells via altering expression of Bcl-2 family proteins, activating the MEK/ERK signaling pathway, and inhibiting aurora B activities [35]. It is reported that by inhibiting heat-shock protein 70 expression, quercetin enhanced chemosensitivity in lung cancer cells [36]. Luteolin is a flavonoid which has been shown to have antitumor effects on lung cancer via degradating EGFR mutation [37], suppressing Raf and PI3K activities [38], regulating ROS-mediated multiple cell signaling pathways [39], activating the p53 pathway [40], a p38/ROS/caspase cascade [41], and MEK/ERK signaling pathway [42]. Kaempferol is a potential radiosensitizer which increased tumor cell killing by radiation via inhibiting PI3K-Akt and ERK pathways as well as the mitochondria apoptosis pathway [43]. It is worth noting that the PI3K-Akt pathway was the identified key pathway of HCT on lung cancer in our study as well. Furthermore, kaempferol and luteolin decreased cell proliferation in lung adenocarcinoma A549 cells by the inhibition of transduction-3 [44]. Beta-sitosterol, a phytosterol induces anticancer properties in different cancers based on different mechanisms [45]. It is indicated that *β*-sitosterol induced G0/G1 cell cycle arrest in NSCLC cells possibly by inactivating the TGF-*β*/Smad2/3/c-Myc pathway [46]. Baicalein exerted a growth inhibitory and prosurvival effect on NSCLC by reducing 12-LOX and VEGF expression and altering the expression of VEGF, FGFR-2, and RB-1 [47]. Another study showed that by inactivating AMPK*α* and MEK/ERK1/2 signaling pathways, increasing FOXO3a and RUNX3 proteins, baicalein inhibited the growth and induced the apoptosis of NSCLC cells [48].

TNF, EGFR, MYC, IL-6, and JUN were identified as major hub genes of HCT on lung cancer. Among them, TNF had the greatest degree in the HCT-major hub genes-disease network, while IL-6, JUN, EGFR, and MYC were shown to associate with the survival of lung cancer patients. These major hub genes could be a promising research area for uncovering the underlying mechanisms of HCT on lung cancer. TNF is a proinflammatory cytokine with multiple functions in homeostatic and pathogenic bioactivities [49]. At the beginning of its discovery, TNF was regarded as an induced factor of necrosis of malignant cells; however, accumulating studies suggest that TNF involves in tumorigenesis, inducing cancer cell survival and proliferation and facilitating tumor metastasis and escape from immunosurveillance [50]. Encoded by TNF, TNF-*α* was associated with apoptosis in the natural killer cell-based therapeutics on lung cancer [51]. Moreover, IL-6 and TNF-*α* could promote metastasis of lung cancer by inducing epithelial-mesenchymal transition in the animal experiment [52].

Encoded by EGFR gene, EGFR is a transmembrane glycoprotein belonging to the ErbB family [53]. EGFR is reported excessively expressed in 85% of NSCLC cells and is associated with a poor prognosis [54]. By regulating downstream signaling pathways, mainly the PI3K/Akt and MAPK, the activated EGFR led to receptor dimerization and tyrosine autophosphorylation which could result in aberrant proliferation in certain cells, such as NSCLC cells [55]. EGFR-mutant lung cancers are one of the most frequent molecularly defined subsets accounting for approximately 40% in East Asians with lung adenocarcinoma, and agents targeted EGFR have been the first-line therapy for NSCLC patients with EGFR mutation [56]. A retrospective cohort analysis (*N* = 285) on OS and characterization of NSCLC patients showed that EGFR-mutant patients had a better OS than nonmutated patients (20.0 vs. 11.0 months, respectively; *P* value: 0.007) [57]. MYC is a protooncogene encodes transcription factors involved in basic cellular pathophysiological processes. Activation of MYC caused abnormal cell proliferation, regression and redifferentiation of cancer cells, and susceptibility to Aurora kinase inhibition in SCLC cells [58]. Transcription factors of MYC attributed to nuclear reprogramming, generation, malignant transformation in artificial stem cells, and enhanced metastatic potential [59]. NSCLC patients with both positive MYC expression and positive PD-L1 expression had poorer OS (HR: 5.223; 95% CI: 2.236 to 12.201; *P* = 0.000) than patients with double-negative expression, which indicated that MYC could be a potential marker for response assessment of immune checkpoint inhibitor therapy [60].

IL-6 is a multifunctional cytokine served as an important regulator of inflammation and involved in protumorigenic activities, including cancer cell proliferation, angiogenesis stimulation, and immune tolerance [61]. The abnormally increased secretion of IL-6 was related to the regulation of growth and metastasis of lung cancer cells, and the activation of the IL-6/AK2/STAT3 pathway enhanced initiation of tumor in lung cancer [62, 63]. Previous studies found that IL-6 blockaded weakened lung cancer tissue construction [64] and lung cancer stem-like cell population could be enriched through IL-6 by inhibiting cell cycle regulators [65]. It is reported that the simultaneous increase in the expression of IL-6 and tumor markers contributed to the worse prognosis of lung cancer patients [66]. As an important member of AP-1 transcription factor family, JUN is involved in growth, metastasis, and drug resistance of cancer [67]. JUN was reported to associate with the acquisition of anchorage independence of lung cancer cell lines, which might contribute to the process of lung carcinogenesis [68]. Though the impact of JUN on the survival of lung cancers remains vague, a study on lung adenocarcinoma cell line (HCC827) showed that increased JUN expression was associated with gefitinib resistance in NSCLC and might lead to a poor prognosis [69].

Our study suggests that the expression level of EGFR, IL-6, JUN, and MYC is associated with the worse OS of NSCLC patients in both univariate survival analysis and multivariate survival analysis after adjusting related clinical features of patients. Additionally, EGFR and MYC expressed significantly higher in LUSC tissues, while IL-6 and JUN expressed significantly lower in both LUSC and LUAD tissues. Therefore, we infer that HCT may benefit the prognosis of NSCLC patients via regulating the expression of these four hub genes. The findings could provide valuable references for future studies on molecular or genetic mechanisms of HCT on lung cancer.

Identified by KEGG pathway enrichment analysis, the MAPK and PI3K-Akt signaling pathways acted as pivotal roles in HCT on lung cancer. Consisted of four signaling families: the MAPK/ERK family, the big MAP kinase-1 (BMK-1), JNK, and p38 signaling families, the MAPK pathway is crucial to cell proliferation, differentiation, migration, and development of drug resistance of malignant cells [70]. The MAPK/ERK pathway can be activated by stimulating growth factor receptors in the cytomembrane. Genetic mutations in the upstream of MAPK/ERK, such as exon 21 mutations in EGFR or del19EGFR, lead to overactivation of the MAPK pathway in the progression of tumorigenesis [71]. In addition, activated by oxidation, genotoxication, osmotic stress, microbial components as well as inflammatory cytokines such as TNF-*α*, the JNK signaling pathway participates in cell apoptosis, tumorigenesis, and inflammation [72]. In the MAPK pathway, MAPK kinase components could phosphorylate diverse target proteins including transcription factors such as c-Jun and c-Myc [73]. C-Jun and c-Myc are encoded by major hub genes of HCT, JUN, and MYC, respectively. The PI3K-Akt signaling pathway was another identified key pathway in our study. Previous studies showed that all major elements of the PI3K pathway mutated or amplified in a wide range of cancers [74]. In NSCLC cells with somatic activated mutations in EGFR, PI3K could be directly activated by EGFR that binds to it [75]. Phosphatidylinositol 3-kinases (PI3Ks) widely participate in the activation of intracellular signaling pathways which regulate processes such as cellular proliferation, adhesion, survival, and motility [76]. A study reported that the PI3K signaling was key to regulate aerobic glycolysis in EGFR-mutant lung adenocarcinoma [77]. In this work, major hub genes including TNF, EGFR, and MYC enriched in the MAPK signaling pathway. EGFR, MYC, and IL-6 enriched in the PI3K-Akt signaling pathway. Notably, EGFR and MYC both involved in these two pathways. Modulating major hub genes via these two pathways may be the main pharmacological mechanisms of HCT in lung cancer treatment.

Based on the comprehensive network pharmacology strategy, key compounds, hub genes, and related pathways of HCT on lung cancer were identified in this study. Expression patterns and clinical significance of major hub genes were also investigated. Limitations existed in this study are listed as follows. Firstly, although molecular mechanisms of HCT on lung cancer were analyzed systematically in the study, yet the insufficiency of experimental evidence limited the conclusion. Further experimental study on HCT, target genes, and its related signaling pathways is needed. Secondly, the findings of the survival analysis indicated that hub genes had an effect on the survival of patients with lung cancer; however, further studies on whether HCT could benefit the prognosis of lung cancer patients by regulating these hub genes are necessary.

## 5. Conclusion

Although HCT has been prescribed to lung cancer patients in the last few decades, the molecular mechanisms of HCT on lung cancer remain unclear. Based on a comprehensive network analysis, our work successfully identified the potential active ingredients, including quercetin, luteolin, kaempferol, beta-sitosterol, and baicalein; major target genes, including IL-6, JUN, EGFR, MYC, and TNF; and potential signaling pathways, including the MAPK and PI3K-Akt signaling pathways. The result of this study suggests that HCT may exert its therapeutic effect on lung cancer by targeting different targets of signaling pathways. The findings could pave the way for advanced research on pharmacological mechanisms of HCT on lung cancer.

## Figures and Tables

**Table 1:** Potential target genes overlap among 9 herbs of HCT.

Herbs	BX (415)	CS (203)	MZC (53)	RS (516)	TD (330)	TLZ (199)	XHC (205)	YXC (371)	ZBM (141)
BX (415)	—	72	48	170	214	76	64	125	59
GCP (203)	72	—	22	85	37	31	28	74	39
MZC (53)	48	22	—	48	49	43	27	37	36
RS (516)	170	85	48	—	126	106	105	261	118
TD (330)	214	37	49	126	—	180	153	171	48
TLZ (199)	76	31	43	106	180	—	161	170	47
XHC (205)	64	28	27	105	153	161	—	175	37
YXC (371)	125	74	37	261	171	170	175	—	82
ZBM (141)	59	39	36	118	48	47	37	82	—

Note: numbers showed within parentheses show total putative targets of each herb. HCT: HeChan tablet; SBX: Sheng Ban Xia (*Pinellia ternate*); GCP: Gan Chan Pi (*Bufo bufo gargarizans Cantor*); MZC: Mao Zhao Cao (*Ranunculus ternatus Thunb*); RS: Ren Shen (*Panax ginseng C. A. Mey*); TD: Tian Dong (*Asparagus cochinchinensis*); TLZ: Ting Li Zi (*Lepidium apetalum Willd*); XHC: Xian He Cao (*Agrimonia pilosa Ledeb*.); YXC: Yu Xing Cao (*Houttuynia cordata Thunb*); ZBM: Zhe Bei Mu (*Fritillaria thunbergii Miq*).

**Table 2:** The quantity of common targets between each herb in HCT and gene targets of lung cancer.

Herbs (target number)	Herb-related targets of lung cancer common target number (507 in total)	Herb-known therapeutic targets of lung cancer common target number (37 in total)
BX (415)	188	13
CS (203)	112	4
MZC (53)	36	4
RS (516)	280	16
TD (330)	199	21
TLZ (199)	166	18
XHC (205)	171	18
YXC (371)	228	17
ZBM (141)	85	12

**Table 3:** Detailed information of major hub genes in PPI network.

Gene names	Degree	Protein names
AKT1	73	RAC-alpha serine/threonine-protein kinase
TP53	65	Cellular tumor antigen p53
MAPK1	63	Mitogen-activated protein kinase
MAPK8	57	Mitogen-activated protein kinase 8
JUN	57	JUN protein
EGFR	55	Epidermal growth factor receptor (epidermal growth factor receptor tyrosine kinase domain)
VEGFA	54	Vascular endothelial growth factor A
IL-6	53	Interleukin-6
INS	51	Insulin
TNF	49	Tumor necrosis factor
MYC	49	V-myc myelocytomatosis viral oncogene homolog

## Data Availability

The datasets used and/or analyzed during the current study are available from the corresponding author on reasonable request.

## Abbreviations

HCT: HeChan tablet
SCLC: Small-cell lung cancer
LUSC: Lung squamous cell carcinoma
LUAD: Lung adenocarcinoma
LCC: Large cell carcinoma
TCMSP: Traditional Chinese Medicine Systems Pharmacology database
BATMAN-TCM: Bioinformatics Analysis Tool for Molecular mechANism of Traditional Chinese Medicine
KEGG: Kyoto Encyclopedia of Genes and Genomes
TTD: Therapeutic Target Database
OMIM: Online Mendelian Inheritance in Man
BP: Biological processes
MF: Molecular function
CC: Cellular component
OS: Overall survival
DFS: Disease-free survival
AKT1: RAC-alpha serine/threonine-protein kinase
TP53: Cellular tumor antigen p53
MAPK1: Mitogen-activated protein kinase
MAPK8: Mitogen-activated protein kinase 8
JUN: JUN protein
EGFR: Epidermal growth factor receptor
VEGFA: Vascular endothelial growth factor A
IL-6: Interleukin-6
INS: Insulin
TNF: Tumor necrosis factor
MYC: V-myc myelocytomatosis viral oncogene homolog
BMK-1: The big MAP kinase-1.

## Supplementary Materials

Supplementary Materials Supplementary file 1: compounds and putative target genes of HCT. Supplementary file 2: 507 common target genes among HCT and related genes of lung cancer. Supplementary file 3: 34 common target genes among HCT and known therapeutic genes of lung cancer. Supplementary file 4: hub gene information of common HCT-related genes of lung cancer PPI network. Supplementary file 5: hub gene information of HCT-known therapeutic genes of lung cancer PPI network, Supplementary file 6: information of hub genes filtered by PPI networks.
